# Effect of a Multispecialty Faculty Handoff Initiative on Safety Culture and Handoff Quality

**DOI:** 10.1097/pq9.0000000000000539

**Published:** 2022-03-30

**Authors:** Katie M. Fitzgerald, Taruna R. Banerjee, Amy J. Starmer, Gregory H. Caplan, Mohammed Alkuwari, Debra F. Hillier, Anne M. Stack

**Affiliations:** From the 1 Department of Pediatrics, Boston Children’s Hospital, Boston, Mass.; 2 Program for Patient Safety and Quality, Boston Children’s Hospital, Boston, Mass.; 3 Department of Pediatrics, Hamad General Hospital, Doha, Qatar

## Abstract

Structured handoffs at transitions of care are vital components of patient safety. A safety culture survey showed that “handoffs and transitions” were among the lowest scoring dimensions at our hospital. We sought to improve physician handoffs and safety culture scores by implementing standardized handoff communication across multiple divisions of an academic pediatric department.

**Methods:**

We used a modified learning collaborative model to implement an I-PASS program, including training, standardized verbal handoff processes, observation and feedback, and sustainment. The setting was the Department of Pediatrics (DoP) within a tertiary academic children’s hospital encompassing 13 clinical divisions. The primary outcome was a change in the DoP staff physician “handoffs and transitions” score on the Agency for Healthcare Quality (AHRQ) Hospital Survey on Patient Safety Culture. Process measures included handoff duration and proportion of handoffs using the complete I-PASS mnemonic.

**Results:**

Five hundred sixty-seven physicians from clinical divisions participated over 14 months. One hundred percent of eligible physicians completed an introductory online I-PASS training module. The “handoffs and transitions” score improved from 46% to 54% from 2018 to 2020. From May 2019 to February 2020, the proportion of observed handoffs with all five elements of the I-PASS mnemonic improved from 62% to 100%, and the duration of handoffs per patient did not change.

**Conclusions:**

We successfully implemented an I-PASS program across an academic department of pediatrics. The departmental staff physician safety culture “handoff and transitions” score improved. The adherence to the I-PASS mnemonic improved. The duration of handoffs did not change over the study period.

## INTRODUCTION

The Joint Commission National Patient Safety Goals emphasize improving communication among hospital staff.^1^ Accurate and complete transfer of patient information and responsibility between medical providers is a fundamental component of healthcare communication. However, the lack of standardization^2,3^ and formal training in medical education^4–6^ limit effective and safe care transfers. Ineffective handoffs are a frequent source of error^7–9^ leading to incorrect and delayed treatment and longer hospital stays.^7,10^ In particular, medically complex patients are vulnerable to inadequate and uncoordinated communication.^11,12^

Prior studies have identified a relationship between safety culture and accurate and complete care transfers.^13,14^ One study reported that effective handoffs are associated with positive perceptions about patient safety.^13^ Another study reported that standardizing handoffs can lead to improved cardiac intensive care unit to acute care unit handoff-related patient safety culture.^14^

There is a growing body of the literature to support structured handoffs. For example, handoff quality in resident-physician-focused programs improved as I-PASS implementation.^15–17^ The mnemonic has a five-item framework: “Illness Severity,” “Patient Summary,” “Action List,” “Situational Awareness and Contingency Planning,” and “Synthesis by Receiver.”^18^ In other work, handoff standardization improved among trainees in large academic centers after I-PASS implementation over a 2- to 3-year period.^19,20^ Moreover, a 30% decrease in adverse events was associated with structured transfers of care.^21,22^ A recent study concluded I-PASS could be successfully applied to various handoff settings, increasing its potential to improve patient safety.^20^ However, there are obstacles to standardizing handoffs. In an earlier study, physicians feared workflow changes and reported limited time given high patient volume.^19^ Also, I-PASS implementation requires significant resources and years to implement in a large academic setting.^19,20^ Contrary to prior findings, a recent study indicated that I-PASS implementation might not need to take multiple years.^17^ To our knowledge, no studies report structured handoff implementation solely among a large group of staff physicians in academic medical centers.

Our institution prioritized handoff improvement after “handoff and transition” scores ranked among the lowest on the Agency for Healthcare Research and Quality (AHRQ) survey in 2018.^23^ Institutional leaders recommended implementing I-PASS. In the Department of Pediatrics (DoP), our largest department, we had a faculty of >500 active staff physicians in 15 clinical divisions. We specifically targeted faculty handoffs to implement I-PASS, as resident orientation incorporated I-PASS training. We aimed to expand beyond shift-to-shift transfers of care, studied previously in our center,^21^ to encourage service handoffs across various pediatric multispecialties and settings. We set a target of 1 year to complete implementation since we felt we could capitalize on the unfavorable AHRQ survey results. Our scope included services that work with medically complex patients. We postulated that implementing I-PASS could improve handoff communication and safety culture scores via this causal pathway.

In anticipation of the hospital-wide effort, the departmental I-PASS team emailed a needs assessment and readiness survey to divisional QI leaders in November 2018 to inform the implementation plan and raise awareness. Among nine survey questions, we included: (1) “Have you had prior divisional I-PASS training?” and (2) “Are you currently using structured handoffs locally?” By November 30, 2018, 12 of 13 physician leaders responded. Three reported having had training, four reported faculty had received a brief overview, and five stated faculty had no prior training. In addition, three leaders reported that their groups were currently using I-PASS in some form, five reported that some were using it, and four reported they were not using I-PASS.

Our primary aim was to improve safety scores for “handoffs and transitions” on the AHRQ Hospital Survey on Patient Safety Culture by 5% through I-PASS implementation. We chose 5% because safety scores are traditionally difficult to improve.^24^ Secondary aims were to achieve attending physician adherence to the I-PASS mnemonic elements to at least 80% and avoid increases in the duration of handoffs.

## METHODS

The setting was the DoP in a freestanding children’s hospital. There are 15 clinical divisions within the department, of which 13 have inpatient services, including the emergency department. There are approximately 567 active staff physicians out of 1,298 hospital-wide. We focused on inpatient and emergency department handoffs because acute care settings are a known source of handoff errors.^25^ The divisions included: Adolescent Medicine, General Pediatrics including Complex Care Services, Emergency Medicine, Endocrinology, Gastroenterology, Genetics, Hematology/Oncology, Immunology including Allergy and Rheumatology, Infectious Diseases, Medical Critical Care, Nephrology, Newborn Intensive Care, and Pulmonary.

**Fig. 1.**
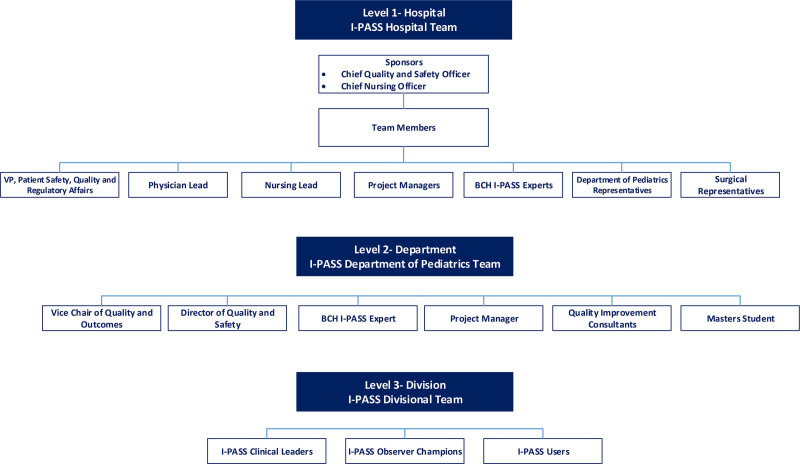
Leadership teams: the I-PASS program led to the creation of leadership teams on the hospital, department, and divisional levels.

There were three levels of leadership for the effort (Fig. 1). Initially, hospital leadership assembled a *hospital-level* I-PASS implementation team. At the *department level*, we were concerned that the hospital I-PASS team would not offer sufficient resources for our ambitious 1-year timeline. Thus, we assembled a departmental-level I-PASS team. Finally, to create an “effector arm” at the local level, we identified *division-level* I-PASS clinical leaders. Divisional teams worked closely with the departmental team, which in turn, worked closely with the hospital team.

## INTERVENTIONS

### Hospital Level

In March 2019, hospital leaders announced a plan to improve handoffs in a newsletter emailed to all clinicians. The newsletter communicated expectations that representatives from all departments attend in-person training and, as needed, attend drop-in office hours and use online project support documents. The hospital I-PASS team assigned providers a mandatory 20-minute online introductory I-PASS training module with a May 2019 deadline. **Materials, Supplemental Digital Content 1,** http://links.lww.com/PQ9/A362, included I-PASS mnemonic pocket cards, literature references, videos of “good and bad” mock handoffs, marketing materials, and an observation data collection tool. In addition, the hospital I-PASS team coordinated the American Board of Pediatrics Maintenance of Certification Part IV (MOC-IV) credit.

### Departmental Level

We chose a learning collaborative framework modeled after the Institute for Healthcare Improvement Breakthrough Series.^26^ We planned to include frequent in-person coaching and encouragement. Our goal was to inform and gain agreement, acceptance, and adherence to the hospital-wide recommendation.^27^ We allowed flexibility in start date, written handoff practices, and type of handoffs (end of service, end of shift, or other) for division leaders to foster acceptance and accommodate workflow differences. We allocated 20 hours/week for I-PASS program planning and implementation during the first 6 months and 10 hours/week for the remaining eight months. Furthermore, the departmental I-PASS team engaged with the hospital I-PASS team at least monthly.

**Fig. 2.**
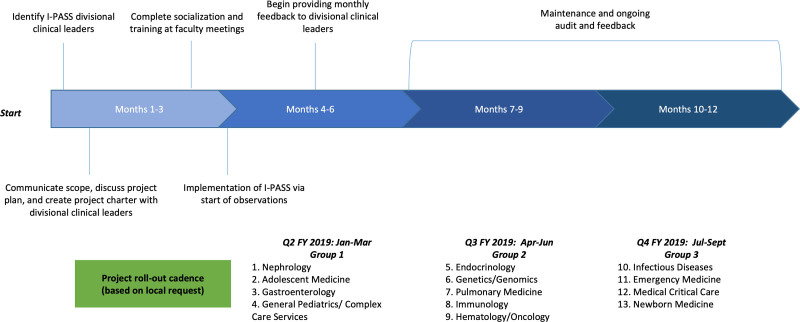
Project timeline: the project timeline supported a rollout cadence specific to each division’s needs.

**Fig. 3.**
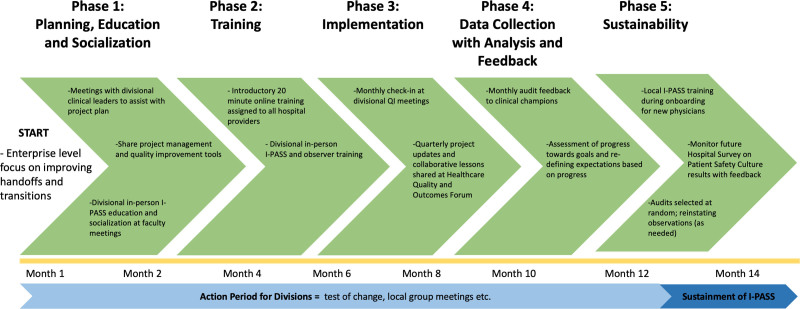
Implementation process: the five identified phases of the implementation process for all divisions.

We sorted 13 divisions into three groups according to local readiness and spaced each group’s rollout at 3-month intervals to conserve resources. We developed a standard implementation process (Fig. 2) with five phases: (1) planning, including a project charter, education, and socialization; (2) training; (3) implementation; (4) data collection with analysis and feedback; and (5) sustainability (Fig. 3). In the planning phase, the departmental I-PASS team met with divisional I-PASS leaders twice within the first month of launch. The first meeting included introducing I-PASS and awareness of enterprise-level leadership expectations. The second meeting entailed the project charter completion and introduction of (1) a sample process map of an “idealized” handoff; (2) an implementation checklist; and (3) faculty communication and training plans.

Next, two divisional faculty meetings were scheduled for physician socialization and education, followed by the identification and training of observer champions. Each division identified local faculty observers and rarely clinical fellows. We used a “train the trainer” model by demonstrating a simulated handoff, providing real-time feedback, and completing the observation data tool, available both on paper and electronically. To reinforce adoption and facilitate learning, we provided project updates, invited divisional leaders to share progress and barriers, and coordinated group discussions at five departmental quality leadership forums from May 2019 to February 2020. We emailed data reports to divisional clinical leaders monthly. The department team reviewed the reports at monthly meetings for reinforcement. Annual reports included progress updates to hospital leadership. Finally, hospital leadership distributed the Hospital Survey on Patient Safety Culture approximately 1 year after the project started.

### Divisional Level

The divisional clinical leaders functioned as I-PASS program organizers with responsibilities that included guidance on local implementation strategy, helping to train local “observer champions,” peer feedback, direct handoff observations, and MOC-IV credit eligibility. They reinforced I-PASS through faculty meetings, email, and daily huddles. Divisions with large faculties employed local administrators to monitor training and observation progress. Some divisions incorporated mnemonic elements into pre-existing written handoff tools. Divisional leaders were also responsible for creating an education and training process for new staff.

### Study of Interventions

For the primary outcome, we analyzed preintervention data for staff physicians on the AHRQ survey administered hospital-wide in 2018 (Hospital Version 1.0) and again during the intervention period in 2020 (a subset of Hospital Version 1.0). We measured the proportion of physicians completing the online training module, the number of completed observations, and mnemonic adherence. We did not record handoff type (ie, shift-to-shift, service change, or across services). The data collection tool in REDCap (Research Electronic Data Capture platform, REDCap Consortium, Nashville, TN) (**see Materials, Supplemental Digital Content 1,** http://links.lww.com/PQ9/A362) included questions about handoff quality for observers to document reinforcing and corrective comments shared verbally in real-time. The data fed into a hospital-wide data dashboard (MicroStrategy, MicroStrategy Incorporated, Tysons Corner, Va.) for analysis and interpretation and refreshed every 24 hours.

### Measures

The primary outcome measure was a change in average score for “handoffs and transitions” on the AHRQ Hospital Survey on Patient Safety Culture for DoP staff physicians from February 2018 to February 2020. The 2018 and 2020 surveys included two questions to assess handoff and transition safety: (1) “Things ‘fall between the cracks’ when transferring patients from one unit to another” and (2) “Important patient care information is often lost during shift changes.” The 2020 survey included a third question, “Communication between departments is effective in this organization.” All questions had the following anchored responses: strongly agree, agree, neither, disagree, and strongly disagree. Process measures included the count of completed observations per division and the proportion of handoffs with adherence to mnemonic elements. Additional process measures included the proportion of staff physicians completing the online training module. Finally, the balancing measure was the observed handoff sessions measured in average minutes per patient.

Hospital-level targets were (1) completion of 10 handoff observations per month or at least 50% of the total number of handoffs performed, whichever was fewer, per division and (2) 80% or greater adherence to the mnemonic by 6 months after the project start date. Once our department met hospital targets, we decreased observation frequency as I-PASS became integrated into practice.

### Analysis

To assess handoff safety culture, we compared the average scores for “handoffs and transitions” that indicated how often physicians selected the two most positive response categories using a 5-point Likert scale (strongly agree, agree, neither, disagree and strongly disagree) on the Hospital Survey on Patient Safety Culture between 2018 and 2020. We utilized run charts to measure the number of observations over time and a control chart for the composite measure of the percent of I-PASS observations with all five elements used consistently.^28^ We applied standard control chart rules to denote special cause variation.^28^ We calculated a simple proportion of physicians completing the online training module. Next, we graphed the median of the duration of handoffs on a run chart. When a handoff session included multiple patients, we estimated the individual handoff time per patient using the time of the entire handoff session. We defined a 2-month baseline period (May–June 2019) as observations began. Finally, we performed a thematic content analysis to analyze the qualitative data from the open-ended questions on the observation form for November 2019–February 2020 (**see Materials, Supplemental Digital Content 1,** http://links.lww.com/PQ9/A362). Thematic coding had an inductive structure to determine themes. We used SAS/STAT software to analyze the data following thematic content analysis completion (Statistical analysis platform, Version 9.4, SAS Institute Inc., Cary, N.C.).

### Ethical Considerations

This work met the criteria for quality improvement and was exempt from IRB review.

## RESULTS

**Fig. 4.**
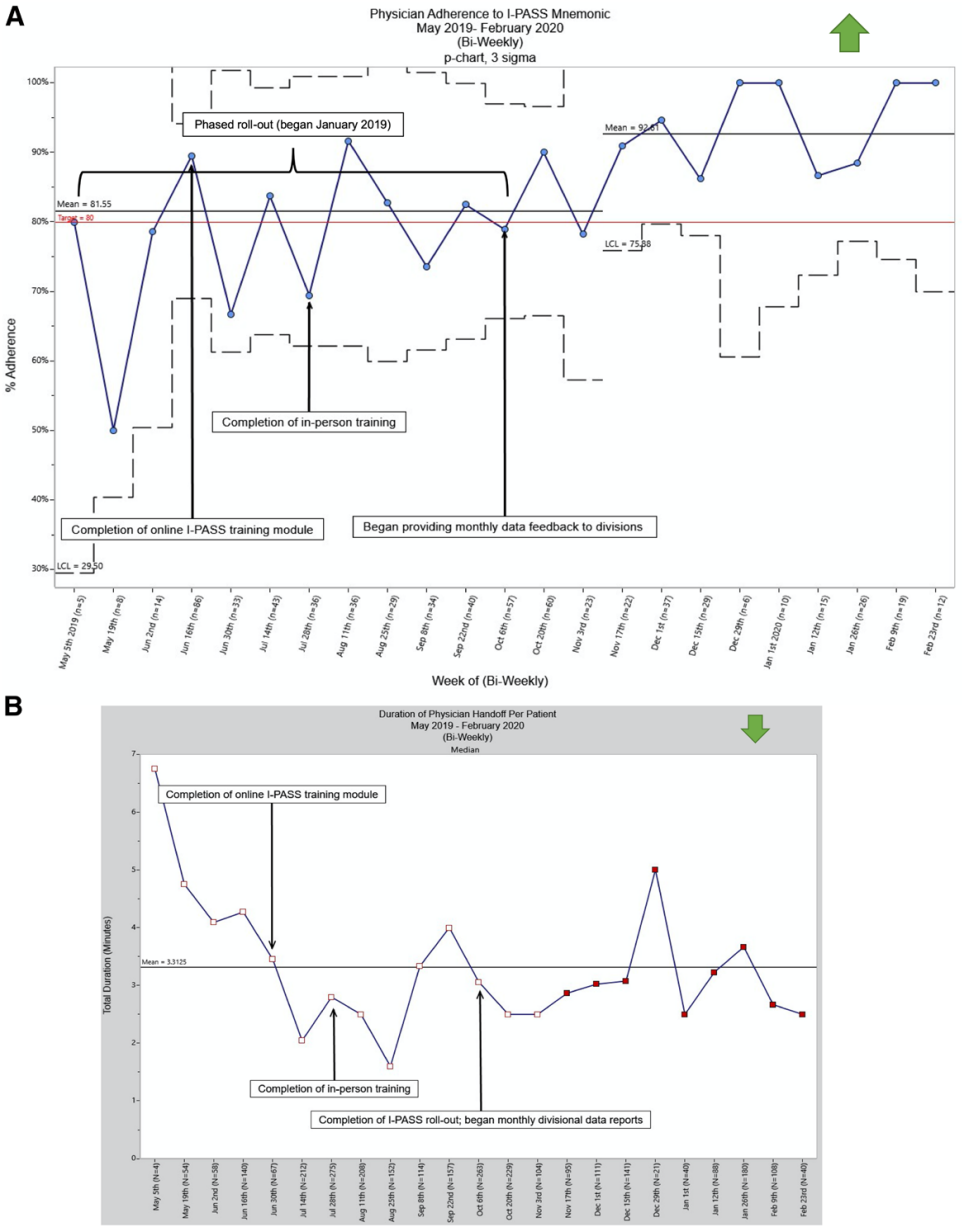
Statistical process control charts for I-PASS improvement initiative. A, Physician adherence to I-PASS mnemonic: physician adherence to the I-PASS mnemonic increased from 62% in May 2019 to 100% in February 2020. B, Duration of physician handoff per patient: duration of physician handoff per patient did not change throughout the intervention period.

For our primary outcome measure, the departmental staff physician score for “handoffs and transitions” increased from 46% to 54% between February 2018 and February 2020. The response rate was 32% (n = 184) in February 2018 and increased to 66% (n = 373) on resurvey in February 2020. All 13 divisions began observations by October 2019. Overall, DoP divisional observers recorded 679 handoff forms over 10 months, averaging 68 completed observations per month from May 2019 to February 2020. The percent with all five elements “consistent” steadily improved with special cause variation, increasing from 62% in May 2019 to 100% by February 2020 (Fig. 4A). In addition, 100% of staff physicians completed the online I-PASS training module by June 2019. Table 1 lists examples of reinforcing and corrective themes for the qualitative analysis. For our balancing measure, the handoff duration did not change (Fig. 4B) throughout the intervention, although there was a shift toward a shorter duration of handoff that was not sustained.

**Table 1.** Overall Frequency of Comment Themes (November 2019–February 2020)

Theme	Count	%	Sample Comment
Reinforcing			
Complete I-PASS mnemonic format*	37	28	“Good summary for both a ‘simple’ and ‘complex’ case. Used the I-PASS acronym tool well to ensure all components used.”
Concise handoff*	32	24	“Very concise patient summaries and plans.”
Clear handoff*	29	22	“Clear summary of patients’ [sic] underlying problem and acute issues provided receiver with understanding that allowed development of clear plan.”
Good synthesis†	23	17	“Consistent thorough receiver synthesis.”
Other‡	44	33	“Good discussion of plan.”
Corrective			
Omission of mnemonic elements‡	38	34	“Illness severity should consistently be stated at the beginning of the sign-out.”
Lack of clarity in mnemonic components*	29	26	“Contingency plan should be more specific.”
None‡	20	18	“No corrections needed.”
Lack of conciseness*	11	10	“Could consider even more succinct summary.”
Other‡	16	14	“Handoff conducted in loud workroom, which may contribute to miscommunications.”

Some comments included more than one theme. “Other” refers to 10 reinforcing and 4 corrective themes each representing <10% of comments.

*Giver-related theme.

†Receiver-related theme.

‡Both.

## DISCUSSION

Using a modified learning collaborative model, we successfully integrated I-PASS across a department of pediatrics in a major children’s hospital. Our current study has several significant findings: first, the structured handoff was implemented among attending physicians rather than residents and included multispecialty pediatric services; second, adoption was associated with improved safety culture scores; and third, implementation resulted in a stable handoff duration throughout our project in support of other findings of a neutral effect.^17^

Miscommunications are a leading cause of medical errors.^7^ Clear and concise handoffs decrease communication-related adverse events.^8^ Previous data from our institution and others have shown that adverse events decreased after I-PASS implementation across a residency program and from ambulatory clinics to the ED^21,22^ communications center.^29^ We found that attending physicians readily accepted structured handoff. Their engagement suggests that the tool is easy to use and adaptable, even for established physicians with diverse patient populations.

Moreover, physician assessment of handoff safety culture for “handoffs and transitions” increased by 8%, exceeding our target of 5%. We attributed the increase in response rates for the hospital survey on patient safety culture from 2018 to 2020 to a hospital-wide campaign to increase physician completion rates. Although it was beyond the scope of our work to measure adverse events related to communication, widespread use of structured handoffs among attending physicians could lead to safer care.

One of the leading barriers to structured handoffs has been the perception that adding the “illness severity” and “synthesis” could add time to lengthy handoffs.^19^ We were able to show that the duration remained stable over the implementation period, as others have reported.^30^

We believe that our proactive outreach fostered acceptance and commitment from providers. In addition, it was flexible enough to allow acceptance by multispecialty services ranging from high acuity care to consultation. We accomplished this by encouraging stakeholder selection of the rollout period, socialization aimed at making handoffs fun, such as showing the “bad handoff” video at faculty meetings, and latitude around a written handoff tool. Mindful of the Model for Understanding Success in Quality and its emphasis on the importance of context, we respected local culture and capabilities.^31^ A benefit of local control may have been that although previous studies focused on shift-to-shift handoffs, attending physicians reported that they expanded I-PASS use to the end of service.

Successful improvement and sustainability of structured handoffs throughout a large healthcare institution require a significant investment in provider training.^32^ We succeeded by staging the project in a cadence that allowed the departmental team to focus on a few divisions at a time. We were then able to learn iteratively from each rollout experience and revise our approach. Although the original timeline for fully embedding I-PASS was 3–4 years, we completed implementation over 1 year. The abbreviated timeline with frequent touchpoints maintained momentum and, we believe, added to our success. This approach may be helpful for the implementation of similar interventions in other settings. We also learned about engagement and content of handoffs through qualitative analysis of open-ended responses.

There are multiple lessons learned as a result of this work. We believe that providing MOC-IV credit helped recruit and retain physician observer champions. We respected the differences in need for guidance and set different expectations for anticipated early adopters versus late adopters. We also were aware of the importance of assessing baseline handoff processes to adapt implementation strategies to align with each division’s unique needs.^8^ Oversight by hospital leadership with regular reporting emphasized the importance of the project.

Our study has several limitations. First, it was performed in a single pediatric hospital and may not be generalizable. Second, we had well-established, highly structured departmental and divisional quality improvement teams in place that other programs may not have. Third, hospital leadership set expectations, and other institutions may not have the same support. Fourth, trained observers completed the observation tool, but self-reporting and the Hawthorne effect may have influenced its completion. Project leaders did not monitor in-person observations, and there was no analysis of the feedback quality to the giver and receiver. Although acknowledging the possibility of the Hawthorne effect, we felt it was essential to be transparent when observing handoffs to allow for immediate feedback. Although observations were not blinded, we aimed to reinforce correct I-PASS use. There was no competition or comparative scoring: the dashboard de-identified the giver, receiver, and observer. Although we had planned to evaluate long-term sustainment, we could not complete this due to COVID-19 disruptions in March 2020. Finally, we targeted staff physicians only. Given the anonymous survey methodology, we cannot compare individual responses from 2018 to 2020 on the AHRQ survey. The differential response rate and other institutional efforts may have contributed to improvement in survey results. Last, divisions did not have the same experience with I-PASS at the start of the project, and those with more experience may have driven aggregate success. Some observers noted a substantial burden in scheduling observation times and completing the observation tool. In the future, one might consider engaging administrators to assist with observations, although establishing cultural norms wherein administrators can offer clinicians formative feedback could prove challenging.

## CONCLUSIONS

Using a modified learning collaborative approach, we successfully implemented an I-PASS program among attending physicians in a department of pediatrics that included multispecialty pediatric services without increasing handoff duration. This methodology was associated with improved safety culture scores. When resources allow, this intensive approach may be helpful in quality improvement work in similar contexts.

## ACKNOWLEDGMENTS

We would like to acknowledge the attending physicians of the Department of Pediatrics.

## DISCLOSURE

AJS holds equity in the I-PASS Patient Safety Institute incorporated in 2016. The I-PASS Patient Safety Institute is a company that seeks to train institutions in best handoff practices and assist in their implementation. The I-PASS Patient Safety Institute was in no way involved in this project. To ensure objectivity, all data were analyzed via a statistical team who do not have involvement with the I-PASS Patient Safety Institute. All analyses were conducted by this statistical team. AJS has received monetary awards, honoraria, and travel reimbursement from multiple academic and professional organizations for teaching and consulting on physician performance and handoffs. The remaining authors have no financial interest to declare in relation to the content of this article.

## Supplementary Material


