# Supplementary material for: Effect of a Multispecialty Faculty Handoff Initiative on Safety Culture and Handoff Quality
Source: Pediatr Qual Saf. 2022 Mar 30;7(2):e539. doi: 10.1097/pq9.0000000000000539 (PMC8970093; doi:10.1097/pq9.0000000000000539)
Supplement: Supplementary file 1 [file pqs-7-e539-s001.pdf]

# I-PASS Handoff Observation Tool

**Date:** ___/___/___ **Start Time:** (HH:MM) _________ **End Time:** (HH:MM) _________

**Handoff Information:**

**Type of Service:** _______________________ **-AND/OR-** Unit: _______________________

**Title of Observer:**

| | | | | | | | | | | | | | |
|---|---|---|---|---|---|---|---|---|---|---|---|---|---|
| Physician | | Fellow/Resident | | RN | | NP | | PA | | QIC | | Other: | |

Observer ID: _______________________ *(Needed for MOC credit)*

**Title of Hand Off Giver:**

| | | | | | | | | | | | | | |
|---|---|---|---|---|---|---|---|---|---|---|---|---|---|
| Physician | | Fellow/Resident | | RN | | NP | | PA | | QIC | | Other: | |

Giver ID: _______________________ *(Please enter if your unit leader/chief will be tracking participation)*

**Title of Hand off Receiver:**

| | | | | | | | | | | | | | |
|---|---|---|---|---|---|---|---|---|---|---|---|---|---|
| Physician | | Fellow/Resident | | RN | | NP | | PA | | QIC | | Other: | |

Receiver ID: _______________________ *(Please enter if your unit leader/chief will be tracking participation)*

---

**Number of patients handed off that you observed:** _____

**I-PASS Handoff Communication** *During the Handoff report, please indicate the following:*

| Observation of Person **GIVING** handoff | Consistently | Not Consistently |
|---|---|---|
| **I** - Was the Illness severity / Acuity of Patient(s) communicated? | | |
| **P** - Was there a patient summary provided? | | |
| **A** - Were action items for the next shift communicated, or did the giver specify "No action items?" | | |
| **S** - Was situational awareness or contingency planning for the patient(s) communicated, or did the giver specify "No anticipated issues"? | | |
| Observation of Person **RECEIVING** handoff | Consistently | Not Consistently |
| **S** - Did the receiver verbalize a synthesis/summarize what was heard? | | |

**Estimate the total number of interruptions that occurred during the handoff session?** 0-2 3-5 >5

*(Ex: Phone calls, Codes, Socialization, etc.)*

---

**Questions/ Comments**

| | Consistently | Not Consistently |
|---|---|---|
| Giver actively engaged with receiver to ensure understanding of patients | | |
| Giver appropriately prioritized key information, concerns, or actions | | |
| To-do list restricted to items that need to be accomplished on next shift | | |
| Used high quality contingency plans with clear if/then format | | |

**Share one positive REINFORCING piece of feedback based on your handoff observation**

_______________________________________________________________________________
_______________________________________________________________________________
_______________________________________________________________________________

**Share one CORRECTIVE piece of feedback based on your handoff observation**

_______________________________________________________________________________
_______________________________________________________________________________
_______________________________________________________________________________
